# Factors that indicate performance on the MoCA 7.3 in healthy adults over 50 years old

**DOI:** 10.1186/s12877-024-05102-1

**Published:** 2024-06-01

**Authors:** César Bugallo-Carrera, Carlos Dosil-Díaz, Arturo X. Pereiro, Luis Anido-Rifón, Manuel Gandoy-Crego

**Affiliations:** 1 Department of Developmental Psychology, University of Santiago de Compostela, Santiago de Compostela, Spain; 2 AtlanTTic, University of Vigo, Vigo, Spain; 3 Department of Psychiatry, Radiology, Public Health, Nursing and Medicine, University of Santiago de Compostela, Santiago de Compostela, Spain

**Keywords:** Aging, MoCA Test performance, Cognitive reserve, Depressive symptoms, MoCA test

## Abstract

Human aging is a physiological, progressive, heterogeneous global process that causes a decline of all body systems, functions, and organs. Throughout this process, cognitive function suffers an incremental decline with broad interindividual variability.

The first objective of this study was to examine the differences in the performance on the MoCA test (v. 7.3) per gender and the relationship between the performance and the variables age, years of schooling, and depressive symptoms .The second objective was to identify factors that may influence the global performance on the MoCA test (v. 7.3) and of the domains orientation, language, memory, attention/calculation, visuospatial and executive function, abstraction, and identification.

A cross-sectional study was carried out in which five hundred seventy-three (573) cognitively healthy adults ≥ 50 years old were included in the study. A sociodemographic questionnaire, the GDS-15 questionnaire to assess depression symptoms and the Spanish version of the MoCA Test (v 7.3) were administered. The evaluations were carried out between the months of January and June 2022. Differences in the MoCA test performance per gender was assessed with Student’s t-test for independent samples. The bivariate Pearson correlation was applied to examine the relationship between total scoring of the MoCA test performance and the variables age, years of schooling, and depressive symptoms. Different linear multiple regression analyses were performed to determine variables that could influence the MoCA test performance.

We found gender-related MoCA Test performance differences. An association between age, years of schooling, and severity of depressive symptoms was observed. Age, years of schooling, and severity of depressive symptoms influence the MoCA Test performance, while gender does not.

## Introduction

Human aging, a physiological progressive heterogeneous global process, causes the decline of all body systems, functions, and organs. Throughout this process, progressive decline in cognitive function occurs, with large interindividual variability [1].

In normal aging, performance deceleration appears in tasks that require divided attention [2]. At the mnesic level, decline in recent episodic memory occurs, possibly due to the effect of the slowing down of speed processing and failures in the processing of information [3]; moreover, errors in working memory, executive functioning, and sensory processing arise [4].

With increasing age, executive deficits also appear, affecting planning, organization, and decision-making, accompanied by a decline in the capacity to learn new concepts; thinking becomes more concrete, with a decrease in the flexibility to perform new abstractions and categorizations [5]. Evidence shows that visuospatial abilities begin to drop from age 80, while visual and perceptual abilities do it from the age of 65 [6].

Studies have shown that age and gender are predictive factors of cognitive performance in aging [7–12]. Some authors report that with the passing of the years women suffer greater cognitive decline in comparison to men [13, 14].

Depression has been receiving increasing interest with regard to cognitive functioning. There are divergences between theoretical and empirical evidences: one the one hand, some authors argue that the presence of depressive symptoms is a risk factor for the development of cognitive deterioration and later progression to dementia [15–19]. On the other hand, other researchers suggest that the decrease in cognitive performance could be explained by the presence of depressive symptoms [20]. More specifically, late onset depression has been more frequently associated to cognitive deterioration rather than early onset depression, late onset depression being more severe and mostly affecting cognition in terms of memory, verbal fluency, visuospatial abilities reaction times, and executive functioning [21, 22]. A third view holds that cognitive performance decline and depression symptoms share common risk factors, which may explain the increase in prevalence of both conditions in older people and the reason of why they are frequently comorbid [23–25].

Cognitive deterioration is not general and homogeneous among the affected individuals; in fact, it has been shown that people with higher level of education show better cognitive performance at old age, which confirms the effect of the variables associated with cognitive reserve in the maintenance of cognitive functioning in adulthood [26, 27]. Similarly, education has been described to be a protective element against cognitive deterioration, associated with the amount of cognitive loss required for the appearance of symptoms [28].

Initially, cognitive reserve was conceived as the brain’s ability to optimize cognitive and functional performance through compensatory mechanisms or the use of alternative cognitive strategies to cope with cerebral insults [29]. In this sense, since cognitive reserve is a theoretical construct, it cannot be measured directly but must be estimated indirectly through sociocultural indicators, such as education or occupation, through the comparison of brain state current with that expected for age, or through functional brain activity using neuroimaging techniques [30].

Besides the effects of age, level of education, mood, and other variables, cognitive functions in advanced stages of life show great interindividual variability and it is difficult to discern between normal and pathological aging; there are no clear limits between them, and sometimes it is very difficult to determine where does one start and the other end. Thus, for an adequate cognitive evaluation it is necessary to know the normal cognitive functioning in an older adult, bearing in mind that cognitive performance may be conditioned by risk factors such as gender, age, state of mind, of by protective factors such the level of education.

In a recent study [31], it has been shown that age, gender, educational level, and depressive symptomatology act as indicators of performance obtained on the Montreal Cognitive Assessment [32]. This instrument is available in multiple languages and scaled for different contexts and populations [33–41], likewise, it has two alternative versions (7.2 and 7.3). Several studies verified the equivalence of the alternative versions with the original version [42–49]. The maximum score is 30; A score equal to or greater than 26 is considered normal with a maximum of 30 points. One point is added if the subject has 12 years or less of education (if the MoCA is less than 30). However, of the alternative versions of the MoCA test, hardly any studies are available.

The purpose of this study was to widen the knowledge on the variables that may be associated with performance on the MoCA 7.3 in cognitively healthy adults ≥ 50 years old, specifically aiming at two objectives. First, to assess cognitive the performance on the MoCA test (v. 7.3) differences based on gender and the relationship between the performance and sociodemographic variables (e.g., age and years of schooling) and emotional variables (e.g., depressive symptoms) in cognitively healthy adults ≥ 50 years old. Second, to estimate the indicative ability of sociodemographic variables and depressive symptoms in the MoCA test 7.3 performance [47] and for each of the seven cognitive domains this instrument assesses (orientation, language, memory, attention/calculation, visuospatial and executive function, abstraction, and identification).

## Materials and methods

A cross-sectional study was carried out in which 573 people residing in Galicia recruited from socio-cultural, professional, and civic associations. The selection of the participants has been carried out by psychologists specialized in psychogerontology through a convenience sample to obtain a sample distributed in a proportionally equivalent manner between age groups (50–59; 60–69; 70–79; and > 80), educational levels (1–4; 5–8; 9–12; and > 13), and gender. The following inclusion criteria were applied: subjects aged ≥ 50 years, without disabling psychiatric disorders, sensorial or motor function impairment, nor doing drugs or under psychoactive medication treatment. Exclusion criteria were absence of cognitive deterioration and illiteracy (participants had to at least know how to read and write). All participants signed an informed consent.

Health professionals (psychologists specialized in psychogerontology) carried out the evaluations at the participant’s home or socio-cultural centers. The following instruments were applied in a partially counterbalanced manner: a sociodemographic questionnaire [48], the Spanish version of the MoCA test (v 7.3), without the correction by education (https://www.mocatest.org), and the 15-item Geriatric Depression Scale (GDS-15) [49]. The evaluations were carried out between the months of January and June 2022.

### Data analysis

Descriptive statistics were computed for each of the sociodemographic variables included in the study. Differences in the MoCA test performance per gender was analyzed using the Student’s t-test for independent samples, homoscedasticity has previously been analyzed through the Levène test. The association between the scoring total of the MoCA test v 7.3 and age, years of schooling, and depressive symptoms was assessed using the bivariate Pearson correlation. To determine which variables showed significant and independent contribution to explain total performance variance (MoCA test) and of each of the seven cognitive domains this test assesses (orientation, language, memory, attention/calculation, visuospatial and executive function, abstraction, and identification), we carried out different stepwise multiple regression analysis. The indicative or independent variables were gender, age, years of schooling, and severity of depressive symptoms. The MoCA test allowed us to determine the effect of each independent variable on the total performance and on the different cognitive domains it evaluates. Prior to carrying out the multiple linear regression analysis in order to guarantee the validity of the model, the assumptions of the multiple linear regression model were verified. The independence of the errors among themselves, that is, the non-self-relationship, was studied with the Durbin-Watson test. To verify the normal distribution of errors, the Kolmogorov-Smirnov test was used. Through White’s test, homoscedasticity was verified, and collinearity has been analyzed through tolerance and the variance inflation factor (FIV). The analyses were carried out with the aid of Statistical Package for Social Sciences (SPSS) statistics for Windows (version 21) (IBM, Armonk, NY).

## Results

Table 1 shows the descriptive statistics of the sociodemographic variables included in this study. We found gender-dependent differences in the total MoCA performance (t = 2.713; gl: 571; *p* < .05) (Student’s t-test) and a statistically significant correlation between the total MoCA performance and the variables age, years of schooling, and severity of depressive symptoms (GDS-15) (Table 2) (*p* < .001). More specifically, there was a statistically significant positive association with years of schooling (*p* < .001) and significantly negative relationships for the variables age and severity of depressive symptoms (*p* < .001).

**Table 1:** Descriptive statistics of socio-demographic variables

Variables	*N* or Mean	% or SD	MoCA score	SD
Gender (female)	358	62.5		
Age	70.06	9.50		
50–59 years	76	13.3	25.43	3.48
60–69 years	199	34.7	24.26	3.88
70–79 years	181	31.6	21.48	4.32
> 80 years	117	20.4	19.41	4.49
Schooling	8.32	3.52		
0–4 years	75	13.1	17.85	3.83
5–8 years	258	45.0	21.24	4.27
9–13 years	138	24.1	24.69	3.34
> 13 years Total GDS-15 (Total score)	102 3.56	17.8 2.51	26.40 22.55	2.53 4.61

Source. Prepared by the authors; SD = Standard Deviation

**Table 2:** Correlations between total cognitive performances based on the Spanish version of the MoCA test (v 7.3) and the variables age, years of schooling, and severity of depressive symptoms

		Age	Schooling	GDS-15
MoCA	Bivariate Pearson correlation	− 0.499**	0.598**	− 0.258**
	Significance (bilateral)	0.000	0.000	0.000
	N	573	573	573

Note: ** *p* < .001

Source. Prepared by the authors

Before carrying out the multiple regression analysis, it was verified that the necessary assumptions were met in all cases to guarantee the validity of the model. Table 3 details statistically significant results for linear multiple regression analyses performed to determine the impact of independent variables on total MoCA test performance and the seven cognitive variables assessed with the MoCA test. Independent variables years of schooling, age, and severity of depressive symptoms were indicators of total MoCA performance, while the variable gender was excluded.

**Table 3:** Significant linear multiple regression analysis of independent variables on total cognitive performance based on the Spanish version of the MoCA test (v 7.3) and of each of the seven cognitive domains evaluated

Factors	B	Beta	t	Significance	95% IC	
					LL	UL
Regression model on total performance (MoCA)						
Years of schooling	0.592	0.453	13.066	0.000	0.503	0.681
Age	− 0.141	− 0.290	-8.444	0.000	− 0.174	− 0.108
Depressive symptoms	− 0.231	− 0.126	-3.954	0.000	− 0.346	− 0.116
Regression model on performance (orientation)						
Age	− 0.016	− 0.269	-6.669	0.000	− 0.021	− 0.011
Depressive symptoms	− 0.026	− 0.117	-2.901	0.004	− 0.044	− 0.009
Regression model on performance (language)						
Years of schooling	0.099	0.360	9.136	0.000	0.078	0.121
Age	− 0.024	− 0.230	-5.879	0.000	− 0.031	− 0.016
Depressive symptoms	− 0.041	− 0.105	-2.900	0.004	− 0.068	− 0.013
Regression model on performance (memory)						
Years of schooling	0.125	0.255	5.926	0.000	0.084	0.167
Age	− 0.031	− 0.172	-4.000	0.000	− 0.047	− 0.016
Regression model on performance (attention/calculation)						
Years of schooling	0.156	0.388	9.637	0.000	0.124	0.188
Age	− 0.021	− 0.140	-3.492	0.001	− 0.033	− 0.009
Depressive symptoms	− 0.070	− 0.124	-3.361	0.001	− 0.111	− 0.029
Regression model on performance (visuospatial and executive function)						
Years of schooling	0.127	0.371	9.565	0.000	0.101	0.153
Age	− 0.034	− 0.266	-6.856	0.000	− 0.044	− 0.024
Regression model on performance (abstraction)						
Years of schooling	0.054	0.336	7.984	0.000	0.041	0.068
Age	− 0.007	− 0.124	-2.969	0.003	− 0.012	− 0.003
Depressive symptoms	− 0.021	− 0.094	-2.439	0.015	− 0.039	− 0.004
Regression model on performance (identification)						
Age	− 0.011	− 0.207	-4.789	0.000	− 0.016	− 0.007
Years of schooling	0.030	0.206	4.764	0.000	0.017	0.042

Note: IC = Interval Confidence; LL = Lower Limit; UL = Upper Limit

Note: *p* < .001

Source. Prepared by the authors

Regarding MoCA test domains test orientation, language, memory, attention/calculation, visuospatial and executive function, abstraction, and identification, we observed that age was the only common indicator for performance for all the domains. Years of schooling was a indicator of performance in all domains except orientation, and the independent variable severity of depressive symptoms a indicator for performance in orientation, language, attention/calculation, and abstraction.

## Discussion

Regarding the first aim of this study, we establish gender-related differences in total MoCA test performance, as well as a negative association between the total MoCA test performance with age and severity of depressive symptoms, and a positive relationship with years of schooling. Our findings are in line with other works that report differences based on gender [13, 14] age [12], depression symptoms [17, 18, 20], and years of schooling [26, 27].

As for our second objective (to estimate the predictive ability of sociodemographic and depression symptoms on global MoCA test performance), age and severity of depression symptoms are negative indicators and years of schooling is a positive indicator for global cognitive performance. On the other hand, gender is not a indicative factor of MoCA test performance.

When we examine the influence ability of each of the study variables on MoCA test performance, age is the only one that acts as a common negative indicator for all the domains examined.

Depressive symptoms are also negative indicators; however, age is a better indicator, as depressive symptoms are unable to explain MoCA Test performance as well as the domains memory, visuospatial/executive function, and identification.

The only positive indicative factor we identify in our study is years of schooling (associated with cognitive reserve). It works as a indicator of good MoCA Test performance in all the assessed domains, except orientation.This indicates that older people with greater cognitive reserve could have more alternative strategies and compensatory mechanisms to achieve more effective and flexible cognitive functioning, with educational level being the main indicator of cognitive reserve [50, 51].

In this work, we identify the factors that can help explain the eventual performance on the MoCA test in healthy older adults and outline a profile of individuals with low performance, who are more vulnerable to suffer cognitive deterioration. With this in mind, designing a protocol to help detect individuals at risk of suffering cognitive deterioration will alert specialists on the need of a follow-up to detect deterioration as early as possible and establish an early treatment [52, 53]. Achieving this would reduce the costs associated to the care of people with dementia [54].

The results obtained in the present study can serve as guidance when carrying out a cognitive evaluation. On the one hand, and prior to the cognitive evaluation, an assessment of the mood of the person we intend to evaluate should be carried out, since the existence of depressive symptoms will negatively condition the results of cognitive performance. Likewise, another factor to take into account when carrying out a cognitive evaluation is the age of the person evaluated since this will negatively condition the results obtained. On the other hand, the cognitive reserve of the person being evaluated must be taken into consideration since this will positively influence the results of the evaluation since it can function as an element that enhances the results of cognitive performance.

Taking into account the above, it would be of interest to have instruments to carry out assessments of cognitive function properly scaled by virtue of the age, depressive symptoms and educational level of the person evaluated, in order to carry out an adequate cognitive assessment.

Further longitudinal studies are needed to assess the keys of cognitive performance in the MoCA Test that help identify which variables have positive and negative effects and determine heterogeneous profiles based on these variables. Likewise, it would be advisable to extend the study to other geographical regions to check whether or not there are differences in the results.

In this original study there are limitations that are accepted by the authors and that make it difficult to generalize the results. One limitation is that referred to the composition of the sample since all the participants come from the same geographical region, another limitation is the use of a cross-sectional design and the possible existence of a cohort effect.

## Data Availability

The datasets used and/or analysed during the current study are available from the corresponding author on reasonable request.

## Abbreviations

GDS-15: Geriatric Depression Scale
MoCA Test (v 7.3): Montreal Cognitive Assessment
FIV: variance inflation factor
SPSS: Statistical Package for Social Sciences
